# Chlorogenic acid promotes mtDNA leakage to enhance cGAS-STING induced anti-tumor immunity via ATF5-mtHSP70-TFAM system

**DOI:** 10.7150/ijbs.136878

**Published:** 2026-08-12

**Authors:** Rui Li, Ping Zhou, Chu-juan Hu, Ge Si, Ling Ren, Jin-jin Cui, Ying-ying He, Yan-xing Han, Jie Zhang, Wen-bin Li, Lu-lu Wang, Jian-dong Jiang

**Affiliations:** 1 State Key Laboratory of Bioactive Substance and Function of Natural Medicines, Institute of Medicinal Biotechnology, Chinese Academy of Medical Sciences & Peking Union Medical College, Beijing, 100050, PR China.; 2 Institute of Materia Medica, Chinese Academy of Medical Sciences & Peking Union Medical College, Beijing, 100050, PR China.; 3 Beijing Key Laboratory of Long-acting Targeted Delivery Technology and Application for Peptide and Nucleic Acid Drugs, Institute of Medicinal Biotechnology, Chinese Academy of Medical Sciences & Peking Union Medical College, Beijing, 100050, PR China.; 4 Jiuzhang Biochemical Engineering Science and Technology Development Co., Ltd, Chengdu, Sichuan, 610041, PR China.; 5 Department of Neuro-oncology, Cancer Center, Beijing Tiantan Hospital, Capital Medical University, Beijing, 100070, PR China.

**Keywords:** chlorogenic acid, mitochondria, TFAM, ATF5, cGAS-STING

## Abstract

Mitochondrial targeting represents a promising antitumor strategy by modulating cell differentiation, metabolic reprogramming, and immune responses. While chlorogenic acid (CGA) has demonstrated the ability to induce tumor cell differentiation and enhance antitumor immunity, the involvement of mitochondrial regulation in these effects remains unclear. This study investigated whether CGA mediates antitumor immune effects through mitochondrial regulation, thereby providing a theoretical framework for natural product-based, mitochondria-targeted therapies. Our findings reveal that CGA inhibits the translocation of mitochondrial transcription factor A (TFAM) into the mitochondria and promotes mtDNA leakage by disrupting the ATF5-mtHSP70 signaling axis. The cytosolic leakage of mtDNA activates the cGAS/STING pathway, triggering the activation of natural killer (NK) cells and cytotoxic T lymphocytes (CTLs), which ultimately facilitates antitumor immunity. In a mouse model, ATF5 knockout enhances cGAS/STING signaling and subsequent immune responses, leading to tumor growth inhibition. These results highlight a novel role of CGA in regulating mitochondrial-associated proteins, positioning it as a potential therapeutic strategy for cancer via the mtDNA-cGAS-STING pathway.

## Introduction

Mitochondria are essential organelles with diverse functions, including energy production, redox balance, cell death regulation, differentiation, and cellular signaling. Mitochondrial dysfunction is implicated in various pathologies, such as metabolic disorders, aging, and cancer. In tumor cells, alterations in mitochondrial function and morphology contribute to excessive cell proliferation and tumor progression. The shift in tumor cell metabolism, characterized by abnormal glucose uptake and lactate production, has long been recognized as aerobic glycolysis, or the “Warburg effect.” However, recent studies have highlighted the critical role of oxidative phosphorylation (OXPHOS) in tumor cell glucose metabolism. Cancer stem cells (CSCs), a subset of cancer cells responsible for tumor growth, metastasis, recurrence, and drug resistance, exhibit mitochondrial dysfunction 1. Growing evidence suggests that CSCs are highly reliant on OXPHOS for energy production, involving increased mitochondrial mass and ATP generation 2, 3. Inhibiting OXPHOS has been shown to induce cancer cell differentiation and suppress tumor growth 4, 5. Pharmacological modulation of mitochondrial function and protein expression is therefore opening new avenues for anti-tumor therapies.

Mitochondria are semi-autonomous organelles containing their own genome (mtDNA), which encodes 13 polypeptides critical for the electron transport chain (ETC) and ATP synthase 6. Mitochondrial transcription factor A (TFAM), encoded by nuclear DNA and transported into the mitochondria, is a key regulator of mtDNA replication, transcription, and mitochondrial biogenesis 7. Previous studies have linked TFAM to cell differentiation, such as its downregulation correlating with upregulation of p21 expression 8, and mitochondrial regulation 9, highlighting its potential as a therapeutic target in oncology. Additionally, increasing evidence supports a role for TFAM-regulated mtDNA in triggering anti-tumor immune responses. Recent findings have shown that loss of TFAM leads to mtDNA leakage into the cytoplasm, activating the cGAS-STING pathway 10, 11.

As a sensor of double-stranded DNA (dsDNA), cGAS recognizes dsDNA to activate the downstream STING pathway, which in turn stimulates TBK1 and IRF3, promoting the secretion of type I interferons (IFNs) and pro-inflammatory cytokines, and recruiting various immune cells 12. In certain cancers, defects in the STING pathway can suppress immune responses, leading to immune evasion. Consequently, a range of STING agonists have been explored as potential cancer therapies, including cyclic dinucleotide (CDN) analogs, non-CDN chemotypes, CDN-loaded exosomes, engineered bacterial vectors containing STING-activating molecules, and small molecule-nucleic acid hybrids 13. These agonists have demonstrated anti-tumor effects either as monotherapies or in combination with other treatments, particularly immune checkpoint inhibitors 14. However, challenges persist with the use of STING agonists, such as toxicity and off-target effects of non-selective agonists, which may induce cytokine release syndrome and systemic inflammation 13, 15.

Natural products have gained recognition for their safety and efficacy in cancer treatment, positioning the identification of indirect STING agonists from natural medicines as a promising therapeutic strategy 16. Due to their chemical structures, natural compounds may activate STING signaling via alternative pathways. Rocaglamide, derived from *Aglaia odorata*, promotes NK cell infiltration and enhances anti-tumor immunity by activating the cGAS-STING pathway through mtDNA in non-small cell lung cancer (NSCLC) 17. Similarly, tetrandrine, derived from *Stephania tetrandra* S. Moore, inhibits NSCLC tumor growth by stimulating the STING/TBK1/IRF3 pathway via dsDNA, and enhances the efficacy of immunotherapy when combined with anti-PD-1 antibodies 18. Based on the activation of the cGAS-STING pathway and its relationship with TFAM and mtDNA leakage, compounds that downregulate TFAM and promote mtDNA release in tumors may act as promising cGAS-STING agonists for anti-tumor therapy.

Chlorogenic acid (CGA), a natural polyphenolic compound, exhibits a range of bioactivities, with its anti-tumor effects supported by clinical trials and experimental research. In a Phase I clinical trial (NCT02728349) for recurrent high-grade glioma, CGA treatment significantly improved overall survival and demonstrated a favorable safety profile 19, and a Phase II clinical trial (NCT03758014) has recently been completed. Our prior research has shown that CGA induces cancer cell differentiation and inhibits cell proliferation via c-Myc SUMOylation 20. Further research revealed that CGA inhibits the phosphorylation of mitochondrial ACAT1 at the Y407 residue, impairing cancer cell proliferation 21. Additionally, CGA suppresses IFN-γ-induced PD-L1 expression, enhances cytotoxic T cell infiltration in tumor tissues, and boosts the anti-tumor efficacy of PD-1 antibodies 22.

Given that differentiated cells, which share markers with senescent cells, exhibit TFAM modulation and mtDNA leakage into the cytoplasm 23. CGA-induced tumor differentiation may involve TFAM regulation and subsequent mtDNA leakage, further enhancing anti-tumor immune responses. This study demonstrates that CGA reduces TFAM translocation into mitochondria by inhibiting the ATF5-mtHSP70 system, leading to mtDNA leakage into the cytoplasm. This, in turn, activates the cGAS-STING pathway, resulting in enhanced NK cell and cytotoxic T lymphocyte (CTL) infiltration, ultimately inhibiting tumor progression. These findings suggest that CGA may serve as a safe and effective STING agonist for clinical cancer treatment.

## Methods and Materials

### Chemicals

Chlorogenic acid (CGA) was derived from the Jiuzhang Biochemical Engineering Science and Technology Development Co., Ltd. (Chengdu, Sichuan, China). CGA was dissolved in normal saline (NS) to prepare a 100 mM stock solution.

### Cell culture

Breast cancer cell line MDA-MB-231 and 4T1, human melanoma cell line A375, human glioma cell line U87MG and TJ905, and human hepatoma cell line Huh7 were from the storage of our laboratory. The MDA-MB-231, A375, TJ905 and Huh7 were cultured in the Dulbecco's Modified Eagle's Medium (Invitrogen, CA, USA) supplemented with 10% fetal bovine serum (FBS) and 1% penicillin/streptomycin (P/S, Invitrogen). The U87MG was cultured in Minimum Essential Medium (Invitrogen) supplemented with 10% FBS and P/S. The 4T1 cell was cultured in RPMI-1640 medium (Invitrogen) supplemented with 10% FBS and 1% P/S. Cells were cultured in a humidified atmosphere containing 5% CO_2_ at 37 °C. For *in vitro* experiments, all cells were seeded into 12-well tissue culture plates at a density of 3×10^5^ cells /well overnight, followed by treatment with different concentrations of CGA (0-200 μM) for 24 h.

### Western blot

Total proteins were extracted from cells or tissues using RIPA lysis buffer or tissue lysis buffer containing Protease & Phosphatase inhibitor (CWBIO, China). Protein concentration was determined using a bicinchoninic acid (BCA) kit. The samples were separated by sodium dodecyl sulfate-polyacrylamide gel electrophoresis (SDS-PAGE) and then transferred to polyvinylidene fluoride (PVDF) membranes, and incubated overnight at 4 °C with primary antibodies against ATF5 (Invitrogen, MA5-32365), mtHSP70 (Invitrogen, MA3-028), cGAS (Proteintech, 29958-1-AP), STING (Proteintech, 19851-1-AP), p21 (Proteintech, 10355-1-AP and 67362-1-Ig), and TFAM (Invitrogen, PA5-80107) respectively. PVDF membranes were washed in TBST three times, followed by incubation with horseradish peroxidase (HRP)-conjugated goat anti-mouse (Beyotime, A0216) and HRP-conjugated goat anti-rabbit IgG (Beyotime, A0208) at room temperature for 2 h. Image Lab 6.0 software was used for data collection and analysis.

### RNA extraction and qRT-PCR

Total RNA was extracted from cells and tissues using the RNAeasyTM kit (Beyotime, R0026, China). 1 μg RNA was retrotranscribed into cDNA using the HiFiScript cDNA synthesis kit (CWBIO, China). The primers used were listed in **Supplementary Table S1**. qRT-PCR was carried out as follows: 95 °C for 10 min, followed by 40 cycles of 95 °C for 15 s and 60 °C for 1 min. The relative gene expression was analyzed using the 2 ^- ∆∆Ct^ method.

### Mitochondrial extraction

Mitochondrial and cytosolic fractions were isolated from cells using the Mitochondria Isolation Kit (ThermoFisher, 89874, USA), according to the manufacturer's instructions. Briefly, cells were harvested and treated with mitochondrial isolation reagents A and B, followed by vortexing for 5 s and incubation on ice. The cells were subsequently added with isolation reagent C and centrifuged at 700 g for 10 min at 4 °C. The supernatant was transferred to a fresh tube and repeatedly centrifuged at 12,000 g for 15 min. The collected supernatant represented the cytosolic fractions, and the pellet was resuspended in 500 μL of isolation buffer C. The final pellet was collected by centrifuging at 12,000 g for 5 min, discarding the supernatant, and then lysing the pellet with RIPA buffer containing Protease & Phosphatase inhibitor.

### Detection of cytosolic mtDNA with staining

Mitochondrial DNA (mtDNA) release into the cytoplasm was measured using immunofluorescent double-label staining. Cells were inoculated in a confocal microscopy dish, cultured for 24 h, and then treated with CGA (200 μM) for 24 h. After several washes, the cells were added with 200 nM Mitotracker Red CMXRos (Yeasen, 40741ES50) and cultured for 15-40 min at 37 °C in the dark. Subsequently, Mitotracker Red CMXRos was removed and the cells were washed twice with PBS, Picogreen dsDNA (Invitrogen, P7581) was added to each cell sample, which was incubated for 45 min at 37 °C in the dark. The Picogreen reagent was discarded, and the cells were stained with Hoechest 33342 (Beyotime, C1082) for 10 min, washed with PBS, and imaged using a fluorescence focal microscope (OLYMPUS FV 3000, Japan). The excitation/emission peaks of MitoTracker Red and PicoGreen dsDNA were 579/599 nm and 502/523 nm, respectively.

### Detection of cytosolic mtDNA with qRT-PCR

Relative level of cytosolic mtDNA was detected by qRT-PCR assay. Cytosolic fractions were prepared from cultured cells using a Mitochondrial Isolation Kit (ThermoFisher, 89874, USA), according to the manufacturer's instructions. The TIANamp Micro DNA Kit (TIANGEN, DP316, China) was used to isolate the mtDNA from the collected cytoplasm. Briefly, Proteinase K was added to the collected cytoplasm, vortexed and incubated with Buffer GB at 56 °C for 10 min. Subsequently, anhydrous ethanol was added and the tube was placed at room temperature for 5 min. The mixed liquid was transferred into the adsorption column. After centrifugation for 1 min at 12,000 r, the waste liquid was discarded and Buffer GD and Buffer PW was added to wash DNA. Finally, the extracted DNA was dissolved using Buffer TB. The mtDNA and nuclear DNA were measured with qRT-PCR using primers that hybridized to sequences in the gene encoding mitochondrial cytochrome c oxidase 1 (mt-Co1) and 18S rDNA, respectively. Primer sequences are provided in **Supplementary Table S1**.

### mtDNA copy number analysis

Total DNA was extracted from cells using a TIANamp Micro DNA Kit (TIANGEN, DP316), and 50 ng of DNA was used for qPCR analysis. The mitochondrial ND1 gene (mtND1) was used to measure mtDNA copy number and was normalized to HGB. Primer sequences are provided in **Supplementary Table S1**.

### ATP and OCR measurement

Oxygen consumption rate (OCR) was measured using Agilent Seahorse XFe96 Analyzer (Santa Clara, CA) and Seahorse XF Cell Mito Stress Test Kit (Agilent, 103015-100). The cell culture medium was replaced with seahorse XF DMEM, including 1 mM sodium pyruvate, 2 mM glutamine and 10 mM glucose. Oligomycin, FCCP and a mixture of rotenone and antimycin A were added to measure basal respiration, maximal respiration, spare respiratory capacity, and ATP-linked respiration, respectively. The OCR values were normalized to cellular protein content.

Intracellular ATP level was measured using an ATP assay kit (Beyotime, S0026). After 24 h of CGA treatment, the cell lysates were collected and centrifuged. ATP detection reagent (100 μL) was added and incubated for 5 min. Then, the sample (20 μL) was added and mixed quickly. Relative light units (RLU) were subsequently measured using a 96 microplate luminometer to evaluate ATP concentrations.

### Multi-immunofluorescence

The location and semiquantitative analysis of cGAS, STING, p21, ATF5, mtHSP70, CD8, IFNγ and GZMB in the tumor tissues and cultured cells were evaluated using immunofluroscent. After dewaxing and rehydration, the sections were immersed in 10% neutral formalin for 10 min, using microwave oven heating for 20 min for antigen retrieval. Non-specific bindings were inhibited with a blocking buffer. Consequently, the sections were incubated with primary antibodies against cGAS (Proteintech, 29958-1-AP), STING (Proteintech, 19851-1-AP), p21 (Proteintech, 10355-1-AP and 28248-1-AP), ATF5 (Invitrogen, MA5-32365), mtHSP70 (Invitrogen, MA3-028), CD8 (Servicebio, GB15068), IFNγ (Proteintech, 29788-1-AP) and GZMB (Proteintech, 13588-1-AP) at room temperature for 1-2 h, respectively. HRP-labeled Polymer anti-rabbit secondary antibody (Servicebio, GB23303) and HRP-labeled Polymer anti-mouse secondary antibody (Servicebio, GB23301) was added for another 30 min. The sections were washed with PBST and incubated with TSA (Tyramide signal amplification) for signal amplication. After dehydration, washing, and sealing with neutral gum, sections were observed and photographed using a scanning fluorescence microscope.

### siRNA transfection

For transient transfection, MDA-MB-231 and A375 cells with 60-80% confluences were transfected with riboFECTTM CP transfection reagent and treated with CGA (200 μM) for 24 h. Cells were collected for multiple color immunofluroscent and qRT-RCR analysis. The target sequences of ATF5 specific siRNAs were obtained from RiboBio Co.

### CRISPR/Cas9 genome editing

ATF5-knockout 4T1 cells were generated via the CRISPR-Cas9 system. Optimal sgRNA target sequences were designed. The cultured cells were seeded at 60% confluence, followed by transfection of Cas9-sgRNA plasmids via electroporation using the GenePulser Xcell system (BioRad) and subsequent selection with puromycin. After isolation of the monoclonal cell populations via infinite dilution, the knockout efficiency was validated via western blotting.

### Animal model

Female Balb/c mice (4 weeks) and male C57BL/6N mice (8 weeks) were obtained from Charles River (Beijing. China). They were housed in a standard specific pathogen-free environment under a 12-h light: dark cycle at 19-21 °C with free access to food pellets and water. All mice were randomly assigned to the control or experimental groups. For the breast cancer model, 1×10^5^ 4T1 cells and ATF5-KO 4T1 cells resuspended in 100 μL of PBS were injected into the mouse mammary fat pad. For the colon cancer model, 2×10^5^ MC38 cells were injected subcutaneously on the back. Mice were injected intraperitoneally with 50 mg/kg of CGA or saline solution daily for 27 days or 18 days. During the experiment, the tumor volume of the mice was measured every 2 days using a digital caliper and calculated using the equation: volume = (length×width^2^)/2. The maximum tumor size allowed is 1500 mm^3^, and all experiments were conducted in accordance with this limit. All the animal experiments were approved by the ethics committee of the Institute of Materia Medica, Chinese Academy of Medical Sciences and Peking Union Medical College (Beijing, China).

### Flow cytometry

Tumor tissues were freshly collected and minced into small pieces, and filtered through a 70 μm strainer. Single-cell suspensions prepared from tumor tissues were subsequently stained and analyzed by flow cytometry. Before antibody staining, all samples were incubated with CD16/CD32 antibody (BioLegend, 101330) to block non-specific staining. For surface staining, single cell suspensions were washed once with MACS buffer, followed by incubation with surface marker antibodies CD45 (BioLegend, 103128), CD3 (BioLegend, 100308), CD8 (BioLegend, 100759), CD49b (Invitrogen, 11-5971-82), and NK1.1 (BioLegend, 156508) for 40 min. For the intracellular staining, cells were fixed and permeabilized by Fixation/Permeabilization Kit (BD, 554714). Cells were incubated with the intracellular antibodies GZMB (Invitrogen, 25-8898-82) and IFNγ (Invitrogen, 48-7311-82) for 30 min. Flow cytometric data were acquired on an Attune NxT acoustic focusing cytometer (ThermoFisher Scientific) and analyzed with FlowJo 10.0.0 software.

### ELISA

Blood samples collected from 4T1 and MC38 tumor bearing mice were centrifuged to obtain serum, the serum was diluted 1:1 with diluent buffer. Based on the recommendations of the mouse ELISA Kits of IFNα (E-EL-M3054, Elabscience, Wuhan, China) and IFNβ (E-EL-M0033, Elabscience, Wuhan, China), the levels of IFNα and IFNβ in the serum could be determined using a microplate photometer at 450 nm (Thermo Scientific Multiskan™ FC).

### Statistics

Data are showed as mean ± SEM. A student's t test and Mann Whitney test were used for two-group comparisons, and a one-way analysis of variance (ANOVA) was used to compare more than two groups. All statistical analyses were performed using GraphPad Prism 9 software, and statistical significance is indicated as *p < 0.05, **p < 0.01, ***p < 0.001. n.s, means no significant difference. All data points represent individual biological replicates.

## Results

### 1. CGA-induced cancer cell differentiation is related to mitochondrial regulation

**Figure 1:**
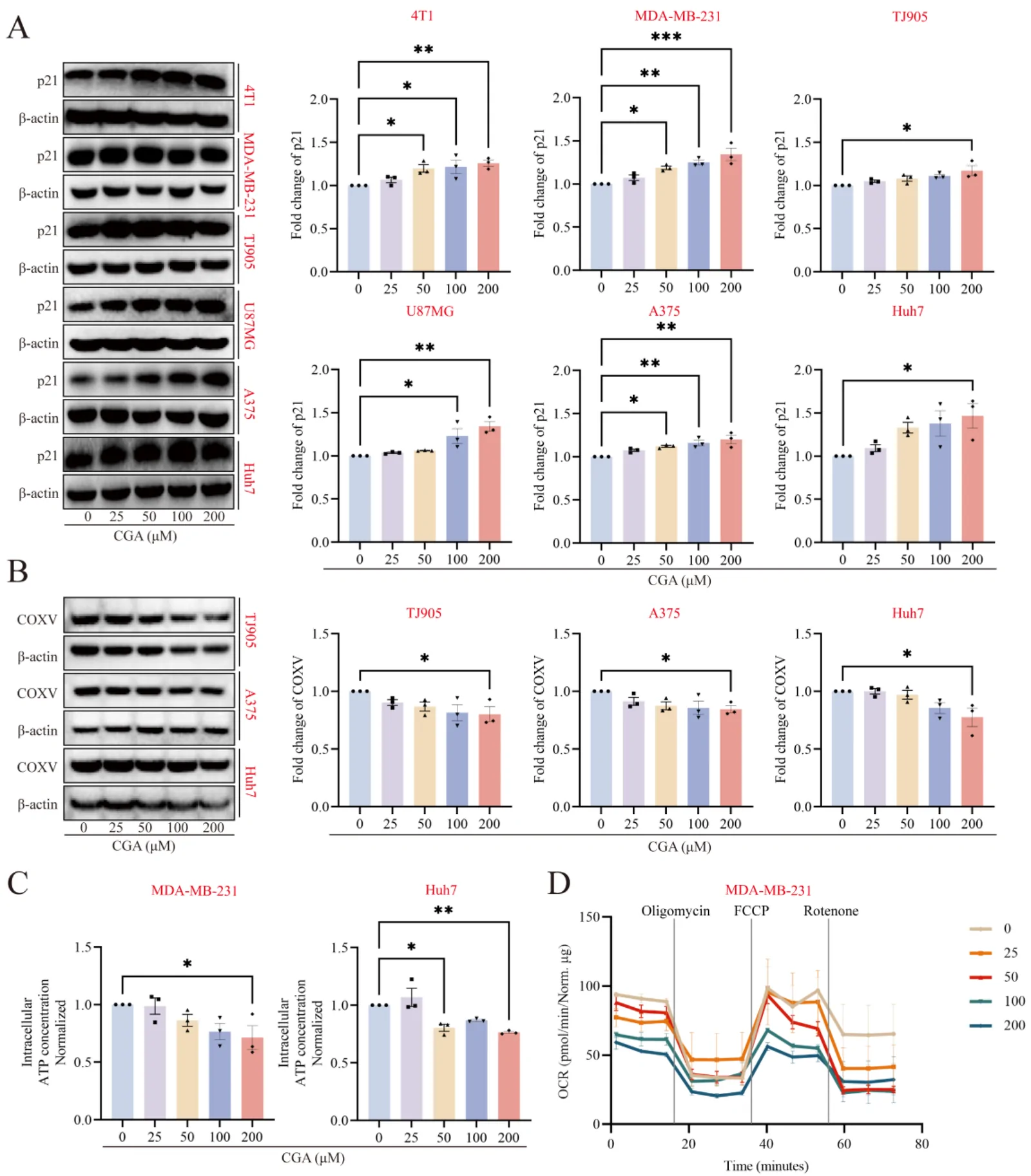
** CGA promoted tumor cells differentiation and modulated mitochondrial function. Breast cancer cell line MDA-MB-231 and 4T1, human melanoma cell line A375, human glioma cell line U87MG and TJ905, and human hepatoma cell line Huh7 were treated with CGA (0, 25, 50, 100, 200 μM) for 24 h. (A)** Western blotting analysis of the protein levels of p21 in tumor cells, β-actin served as the loading control. **(B)** Western blotting analysis of the protein levels of COXV in the indicated cell lines. β-actin served as the loading control. **(C)** Measurement of ATP levels in MDA-MB-231 and Huh7 cells. **(D)** Measurement of the mitochondrial oxygen consumption ratio (OCR) of MDA-MB-231 cells treated with CGA for 24 h. Data are presented as mean ± SEM (n = 3), analyzed using one-way ANOVA. *p < 0.05, **p < 0.01, ***p < 0.001. CGA: chlorogenic acid.

In our previous study, CGA was identified as a cancer differentiation marker, demonstrating upregulation of p21 expression. In the current work, a significant, dose-dependent increase in p21 expression was observed across six different cancer cell lines upon CGA treatment (200 μM for 24 hours; p < 0.01, 0.001, 0.05, 0.01, 0.01, 0.05, respectively) (**Fig. 1A**). Since cancer differentiation can suppress cell proliferation and disrupt metabolic processes related to mitochondrial regulation, mitochondrial function was systematically assessed by measuring cytochrome c oxidase subunit V (COXV) protein expression, oxygen consumption rate (OCR), mitochondrial DNA (mtDNA) copy number, and intracellular ATP levels. COXV, a critical component of the mitochondrial inner-membrane ETC, is essential for OXPHOS; its pharmacological inhibition reduces ATP synthesis and cellular energy supply. As shown in **Fig. 1B**, CGA treatment downregulated COXV expression in three cancer cell lines by 19.9%, 15.6%, and 22.6% (200 μM for 24 hours; p < 0.05, 0.05, 0.05, respectively).

Subsequently, ATP levels were significantly reduced in two cancer cell lines following CGA treatment (**Fig. 1C**, 200 μM for 24 hours; p < 0.05, 0.01, respectively). Since mitochondrial OXPHOS is the primary process for ATP production, OCR was measured using a Seahorse assay after CGA treatment. As shown in **Fig. 1D**, both basal and maximal OCR were inhibited by CGA (200 μM), which likely correlates with the reduced ATP content. Changes in mtDNA copy number reflect mitochondrial OXPHOS activity. Measurement of mtDNA copy number in A375 and Huh7 cells revealed a significant decrease in mtDNA levels following CGA treatment (200 μM for 24 hours; p < 0.01, 0.05, respectively) (**Supplementary Fig. S1**). These mitochondrial function alterations suggest that CGA-induced cancer differentiation may be associated with the inhibition of mitochondrial function.

### 2. CGA reduced TFAM expression in mitochondria, leading to mtDNA leakage and cGAS-STING pathway activation

**Figure 2:**
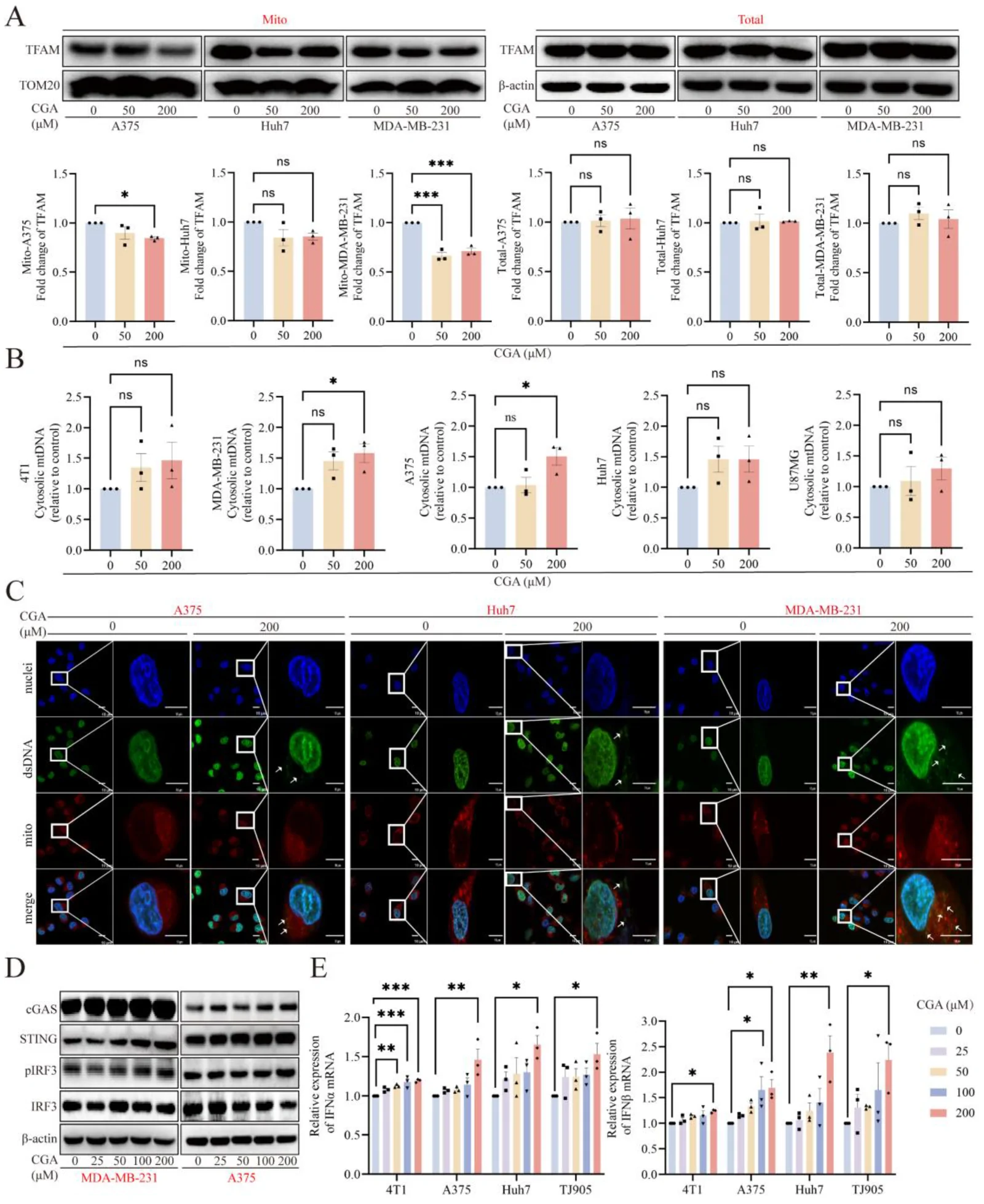
** CGA downregulated the protein expression of TFAM in mitochondria and induced release of mtDNA to initiate cGAS/STING activation. (A)** Western blotting analysis of the protein levels of TFAM in whole cell extract and mitochondria in A375, Huh7, and MDA-MB-231 cell lines. Fractionation fidelity was verified by detection of TOM20 or β-actin. **(B)** qRT-PCR quantification of the levels of mtDNA present in the cytosolic fraction of 4T1, MDA-MB-231, A375, Huh7 and U87MG cells. **(C)** Confocal microscopy images of Double-stranded DNA (green) and MitoTracker (red) in A375, Huh7 and MDA-MB-231 cells. The nucleus was stained with Hoechest33342. Scale bars, 10 μm. **(D)** Western blotting analysis of the protein levels of cGAS, STING, pIRF3, and IRF3 in the indicated cell lines. β-actin served as the loading control. **(E)** qRT-PCR analysis of the mRNA levels of IFNα and IFNβ in the tumor cells. GAPDH served as the loading control. Data are presented as mean ± SEM (n = 3), analyzed using one-way ANOVA. *p < 0.05, **p < 0.01, ***p < 0.001, ns, no significance. TFAM: transcription factor A; dsDNA: double stranded DNA.

TFAM, encoded by nuclear genes and transported to the mitochondria, plays a key role in the transcription and replication of mtDNA and is also involved in the regulation of the mitochondrial respiratory chain. Analysis of TFAM expression in patients with glioblastoma (GBM) revealed significantly higher expression levels compared to normal controls (**Supplementary Fig. S2**). Consequently, the expression of TFAM in the mitochondria was investigated. As shown in** Fig. 2A**, CGA administration significantly inhibited TFAM expression in mitochondria in three cancer cell lines (vs. untreated; 200 μM; p < 0.05, ns, 0.001, respectively), whereas the total TFAM expression remained unchanged. Given that reduced mitochondrial TFAM may facilitate mtDNA leakage into the cytoplasm, the relative mtDNA levels in the cytoplasm were assessed using qRT-PCR. As shown in **Fig. 2B**, CGA treatment significantly increased cytoplasmic mtDNA levels by 1.5-, 1.6-, 1.5-, 1.5-, and 1.3-fold in 4T1, MDA-MB-231, A375, Huh7, and U87MG cell lines, respectively (200 μM; p < ns, 0.05, 0.05, ns, ns). To visualize cytosolic mtDNA directly, this study employed laser confocal microscopy, staining dsDNA with Picogreen and labeling mitochondria with Mitotracker. As shown in **Fig. 2C**, mtDNA in the cytoplasm, distinct from mitochondria (indicated by white arrows), was more pronounced in CGA-treated cells compared to the untreated group in three cancer cell lines.

Since the cGAS-STING pathway can be activated by cytosolic dsDNA, the released mtDNA may act as a potent cGAS-STING agonist 24. Following the increase in cytosolic mtDNA, the expression of cGAS and STING proteins was examined in all cancer cell lines. CGA treatment significantly upregulated cGAS expression in 4T1, MDA-MB-231, A375, U87MG, and Huh7 cell lines (200 μM for 24 hours; p < 0.001, 0.05, 0.05, 0.01, 0.05, respectively) (**Fig. 2D and Supplementary Fig. S3**). Additionally, STING expression was upregulated in 4T1, MDA-MB-231, A375, and TJ905 cell lines with fold changes of 1.5-, 1.3-, 1.2-, and 1.4-, respectively (p < 0.001, 0.01, 0.01, 0.05) (**Fig. 2D and Supplementary Fig. S3**). This study further assessed the expression of pIRF3, a downstream effector of STING signaling, which was significantly upregulated (**Fig. 2D and Supplementary Fig. S3**). Since the cGAS-STING pathway stimulates innate immune responses, the mRNA levels of IFN-α and IFN-β were measured following CGA treatment. As shown in **Fig. 2E**, CGA treatment (200 μM) upregulated IFNα and IFNβ mRNA expression in four cancer cell lines (IFNα: p < 0.001, 0.01, 0.05, 0.05; IFNβ: p < 0.05, 0.05, 0.01, 0.05, respectively). These results suggest that CGA regulates mitochondrial function by inhibiting TFAM in the mitochondria, leading to mtDNA release into the cytoplasm. This, in turn, activates the cGAS-STING pathway, potentially stimulating anti-tumor immunity.

### 3. CGA reduced TFAM in mitochondria through inhibition of ATF5 and the mtHSP70 system

**Figure 3:**
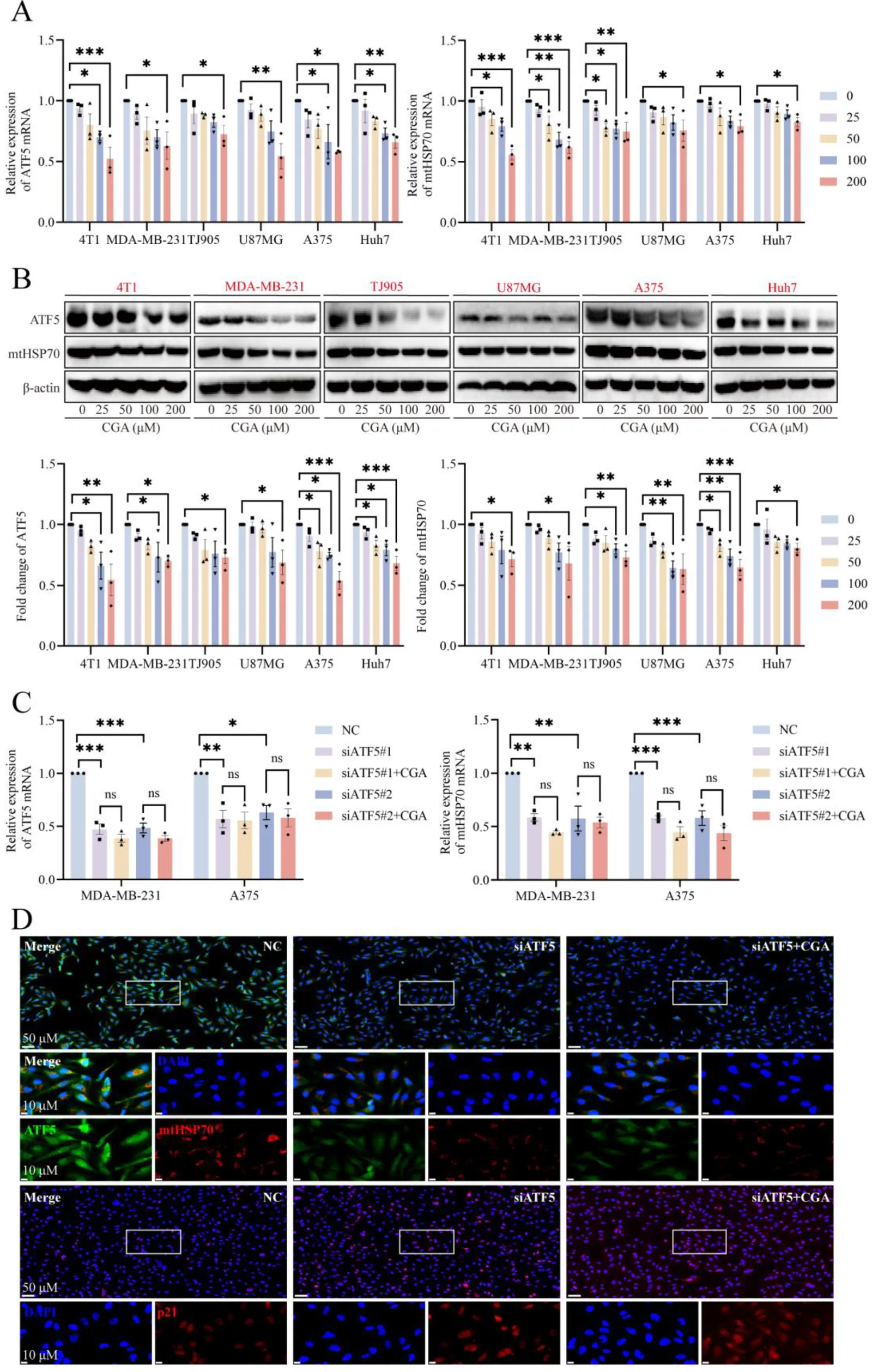
** CGA inhibited the expressions of ATF5 and mtHSP70. (A)** qRT-PCR analysis of the mRNA levels of ATF5 and mtHSP70 in the tumor cells. **(B)** Western blotting analysis of the protein levels of ATF5 and mtHSP70 in the indicated cell lines. **(C)** qRT-PCR analysis of the mRNA levels of ATF5 and mtHSP70 in MDA-MB-231 and A375 cells with knockdown of ATF5. **(D)** Representative images of immunofluorescence staining for ATF5, mtHSP70 and p21 in MDA-MB-231 cells with knockdown of ATF5. The nucleus was stained with DAPI. Scale bar, 50 μm and 10 μm. Data are presented as mean ± SEM (n = 3), analyzed using one-way ANOVA. *p < 0.05, **p < 0.01, ***p < 0.001, ns, no significance.

The translocation of TFAM from the cytoplasm to the mitochondria is likely mediated by the TOM and TIM complexes. Once in the mitochondria, TFAM interacts with mtHSP70, a key chaperone protein, ensuring its proper folding and functionality 25. To investigate this process, the expression of mtHSP70 at both the mRNA and protein levels was measured following CGA treatment. As shown in **Fig. 3A** and **3B**, CGA administration significantly suppressed mtHSP70 expression in six cancer cell lines, suggesting that inhibition of mtHSP70 may contribute to the reduced mitochondrial TFAM levels. ATF5, a key regulator of the mitochondrial unfolded protein response, also functions as a transcription factor for mitochondrial chaperone genes, including mtHSP70, HSP60, and LONP1 26. Detection of ATF5 expression in six cancer cell lines revealed that CGA (200 μM) decreased ATF5 levels at both the mRNA and protein levels, potentially affecting the expression of mtHSP70. To further investigate whether the effects of CGA were linked to ATF5 and mtHSP70 regulation, two different siRNAs targeting ATF5 were transfected into MDA-MB-231 and A375 cell lines to knock down ATF5. As shown in **Fig. 3C**, siATF5s effectively reduced ATF5 mRNA expression in both cell lines (p < 0.001, 0.01, respectively), and CGA-induced decreases in ATF5 were reversed. A similar pattern was observed for mtHSP70 mRNA expression. Immunofluorescence assays were then performed to assess protein-level changes. Analysis of mean fluorescence intensity (MFI) revealed that knocking down ATF5 prevented significant changes in the expression of either ATF5 or mtHSP70 following CGA treatment (**Fig. 3D and Supplementary Fig. S4**). Additionally, increased p21 expression indicated that CGA-induced differentiation might be linked to ATF5 downregulation. These results demonstrate that CGA-induced inhibition of ATF5 leads to a reduction in mtHSP70 levels, which in turn decreases mitochondrial TFAM expression.

### 4. CGA inhibited tumor growth in two tumor xenograft models

**Figure 4:**
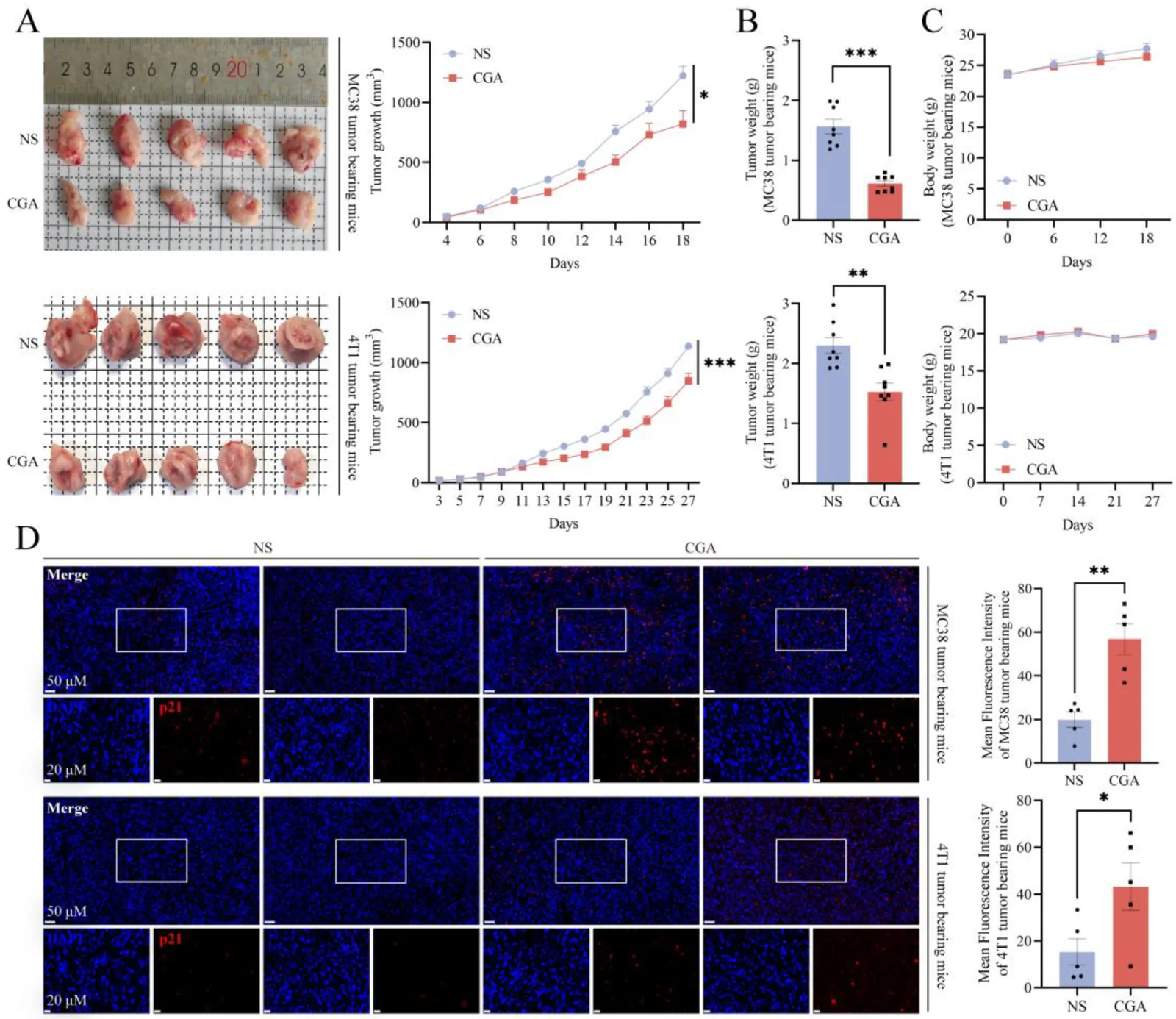
** CGA suppressed tumor growth *in vivo*.** Murine colon carcinoma MC38 cells (2×10^5^ cells per mouse) were subcutaneously injected into the back of C57BL/6N mice (n = 8 for each group). Murine breast cancer cell 4T1 cells (1×10^5^ cells per mouse) were injected into the mammary fatty pad of the Balb/c mice (n=8 for each group). CGA (50 mg/kg) was injected intraperitoneally once a day for 18 or 27 days. Tumor volume was measured starting from 3 or 4 days after inoculation. **(A)** The representative image of excised tumors and tumor growth analysis of MC38 and 4T1 xenografts. Tumor weight **(B)** and body weight **(C)** of MC38 and 4T1 tumor-bearing mice. **(D)** Representative images of immunofluorescence staining for p21 in the MC38 and 4T1 xenograft tumors. Scale bar, 50 μm and 20 μm. Data are presented as mean ± SEM (n = 5 or 8), analyzed using Student's *t*-test. *p < 0.05, **p < 0.01, ***p < 0.001, NS: normal saline.

Previous studies have demonstrated that CGA inhibits tumor growth in various animal tumor models. In this study, two tumor-bearing mouse models were established to assess the antitumor efficacy of CGA (50 mg/kg, i.p. for 18 and 27 days). In the MC38 tumor xenograft model, representing a “Hot” tumor, CGA significantly inhibited tumor growth with a 32.9% inhibition rate (CGA vs. NS, n = 8, p < 0.05) (**Fig. 4A**). A similar inhibitory effect was observed in the 4T1 tumor xenograft model, considered an “Altered” tumor model, with a 25.5% inhibition rate (CGA vs. NS, n = 8, p < 0.001) (**Fig. 4A**). Additionally, tumor weights in both models were significantly reduced by 60.9% (MC38) and 33.6% (4T1) following CGA treatment (CGA vs. NS, p < 0.001, 0.01) (**Fig. 4B**). Body weight changes in both animal models were not significant during the treatment period (**Fig. 4C**), suggesting the safety of CGA administration. Since p21 expression serves as a marker of tumor differentiation, immunofluorescence assays were conducted to evaluate p21 levels in tumor tissues. As shown in** Fig. 4D**, CGA treatment significantly increased p21 expression in tumor tissues, with MFI values increased to 2.9- and 2.8-fold in the MC38 and 4T1 models, respectively (CGA vs. NS, n = 5, p < 0.01, 0.05). These results confirm the antitumor efficacy of CGA in both tumor xenograft models.

### 5. The expression of the ATF5-mtHSP70 axis was inhibited by CGA treatment *in vivo*

**Figure 5:**
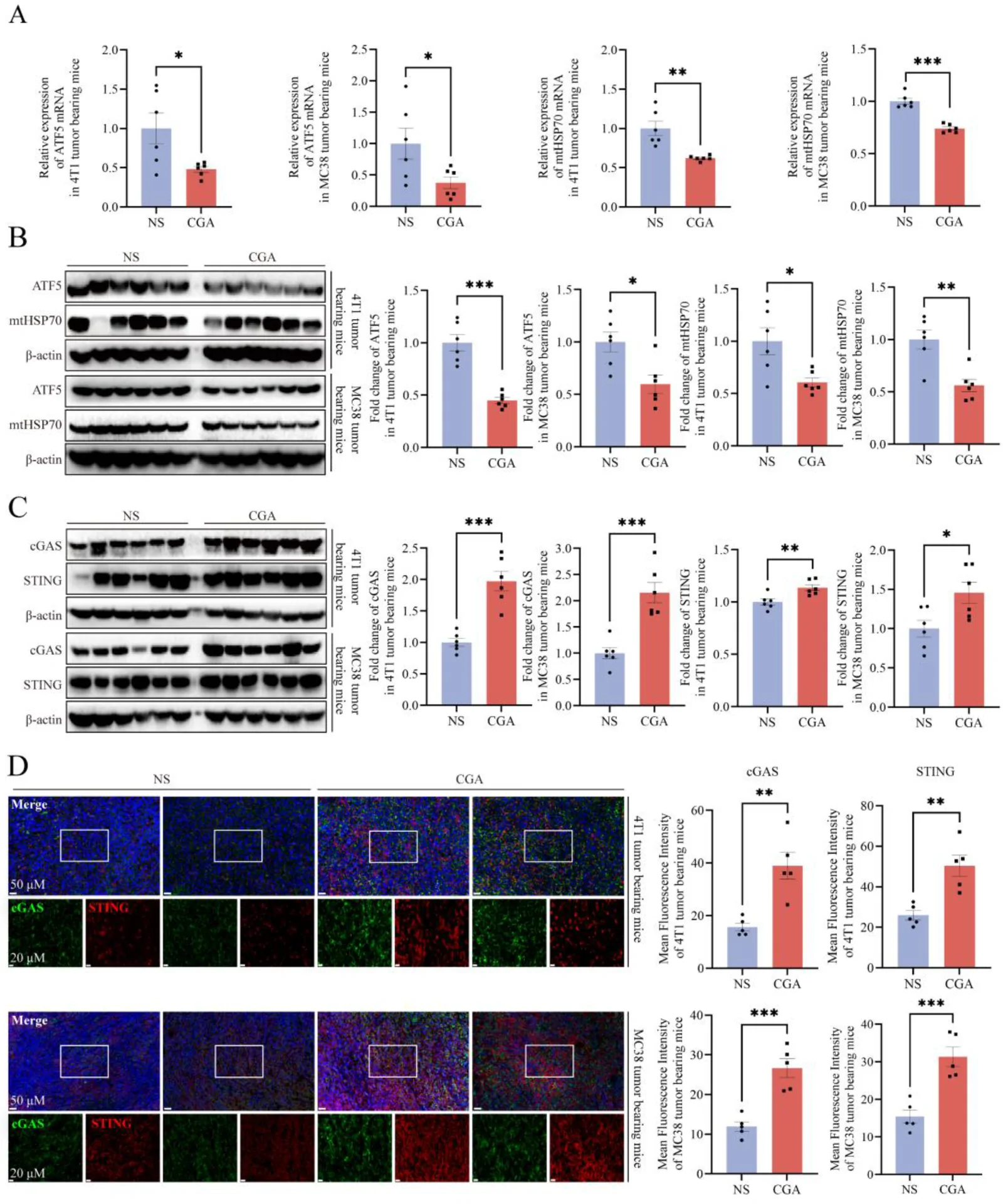
** CGA inhibited ATF5-mtHSP70 axis *in vivo*. (A)** Quantification of mRNA levels of ATF5 and mtHSP70 in 4T1 and MC38 tumor tissues. **(B)** Western blotting analysis of the protein levels of ATF5 and mtHSP70 in tumor tissues. **(C)** Western blotting analysis of the protein levels of cGAS and STING in tumor tissues. **(D)** Representative images of immunofluorescence staining for cGAS (green) and STING (red) in tumor tissues. Scale bar, 50 μm and 20 μm. Data are presented as mean ± SEM (n = 5 or 6), analyzed using Student's *t*-test. *p < 0.05, **p < 0.01, ***p < 0.001.

Following the *in vitro* downregulation of ATF5 and mtHSP70 by CGA, the expression levels of these proteins were assessed in tumor tissues from two models. As shown in **Fig. 5A** and **5B**, CGA treatment reduced the mRNA expression of ATF5 and mtHSP70 (CGA vs. NS, n = 6, p < 0.05, 0.01 for 4T1 tumor model, p < 0.05, 0.001 for MC38 tumor model, respectively). Additionally, the protein levels of ATF5 and mtHSP70 were decreased by 55.1% and 39.4% (CGA vs. NS, n = 6, p < 0.001, 0.05 for 4T1 model, respectively) and 40.2% and 43.9% (CGA vs. NS, n = 6, p < 0.05, 0.01 for MC38 model, respectively). Given that inhibition of ATF5 and mtHSP70 may activate the cGAS-STING signaling pathway, the expression of cGAS and STING was analyzed in both tumor models. CGA treatment significantly upregulated cGAS and STING expression by 2.0- and 1.1-fold in the 4T1 model (CGA vs. NS, n = 6, p < 0.001, 0.01, respectively) and 2.2- and 1.5-fold in the MC38 model (CGA vs. NS, n = 6, p < 0.001, 0.05, respectively) (**Fig. 5C**). Immunofluorescence assays confirmed the upregulation of cGAS and STING following CGA treatment (**Fig. 5D**). Moreover, the mRNA levels of IFN-α and IFN-β were significantly increased after CGA administration (**Supplementary Fig. S5**). However, no significant increase was observed in serum levels of IFN-α and IFN-β as measured by ELISA (**Supplementary Fig. S6**).

**Figure 6:**
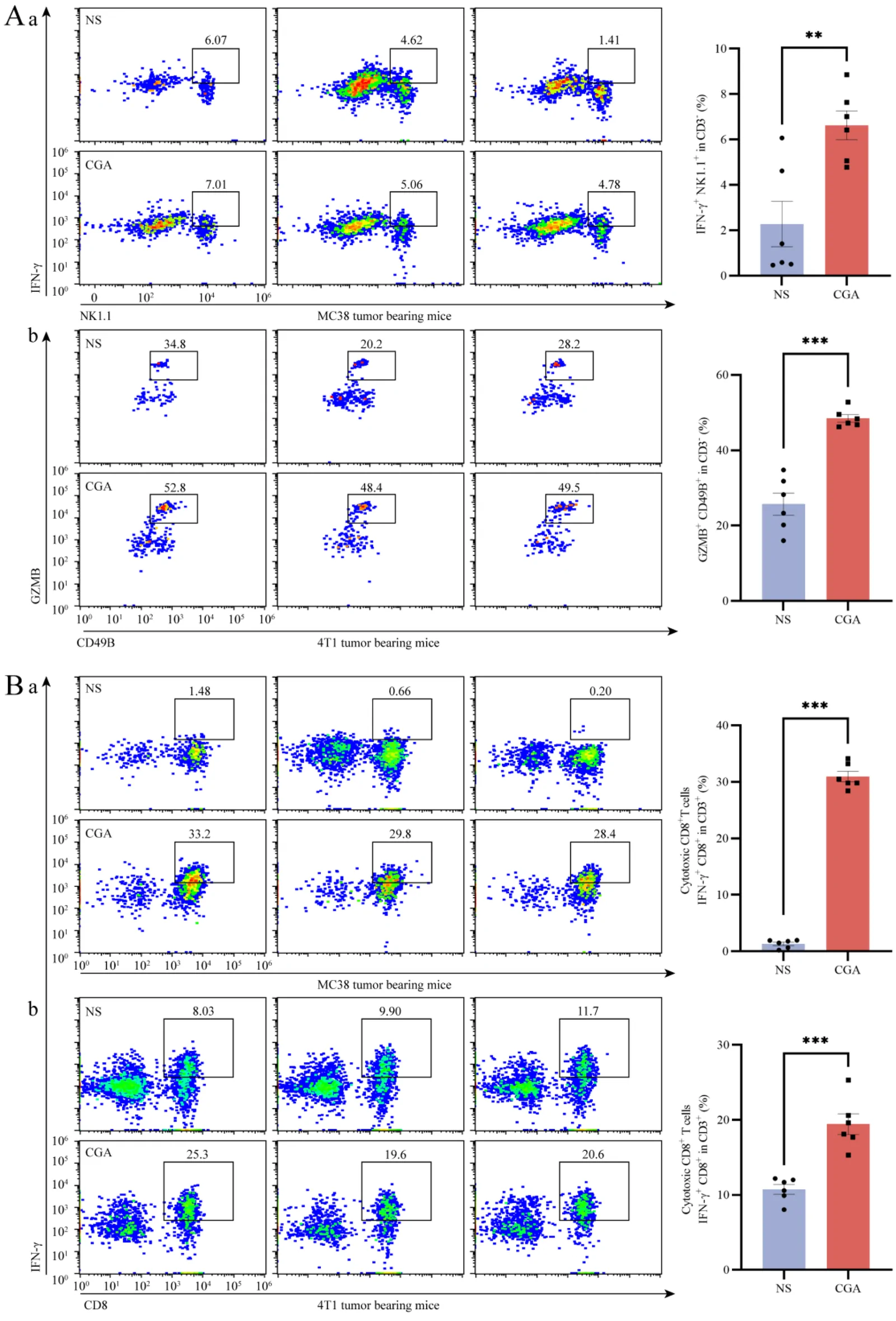
** CGA boosted the infiltration of cytotoxic T lymphocyte and activated NK cells in tumor tissues.** Cytotoxic T lymphocytes (CTLs) and natural killer (NK) cells were isolated from fresh tumor tissues to generate a single-cell suspension for flow cytometry analysis. **(A)** Representative flow cytometer profiles and proportions of activated NK cells (IFN-γ+NK1.1+CD3- or GZMB+CD49B+CD3-). **(B)** Representative flow cytometer profiles and proportions of CTLs (IFN-γ+CD8+CD3+) in MC38 and 4T1 tumor bearing mice. Data are presented as mean ± SEM (n = 6), analyzed using Student's *t*-test or Mann Whitney test. **p < 0.01, ***p < 0.001. IFNγ: interferon-γ; GZMB: granzyme B.

Given that the activation of the cGAS-STING pathway promotes anti-tumor immunity, the populations of tumor-infiltrating NK cells and CTLs—critical players in immune surveillance—were analyzed by flow cytometry. Activated NK cells were identified as IFN-γ+ NK1.1+ or GZMB+ CD49B+ in the CD3- cell population. In the MC38 tumor model, the proportion of IFN-γ+ NK1.1+ NK cells increased from 2.3% to 6.6% after CGA treatment (CGA vs. NS, n = 6, p < 0.01) (**Fig. 6Aa**). A similar increase was observed in the 4T1 tumor model, where the proportion of NK cells (GZMB+ CD49B+) rose from 25.7% to 48.5% with CGA treatment (CGA vs. NS, n = 6, p < 0.001) (**Fig. 6Ab**). Tumor-infiltrating CTLs were marked by IFN-γ+ CD8+ in the CD3+ cell population. In both the MC38 and 4T1 models, CGA treatment significantly enhanced CTL infiltration. As shown in **Fig. 6Ba**, the proportion of IFN-γ+ CD8+ T cells in the MC38 model increased from 1.3% to 31.0% (CGA vs. NS, n = 6, p < 0.001), while in the 4T1 model, the proportion rose from 10.7% to 19.4% (CGA vs. NS, n = 6, p < 0.001) (**Fig. 6Bb**). These results demonstrate that CGA treatment suppresses the expression of ATF5 and mtHSP70 *in vivo*, leading to the activation of the cGAS-STING pathway and the subsequent stimulation of anti-tumor immune responses.

### 6. The anti-tumor effect of CGA was associated with the suppression of ATF5

**Figure 7:**
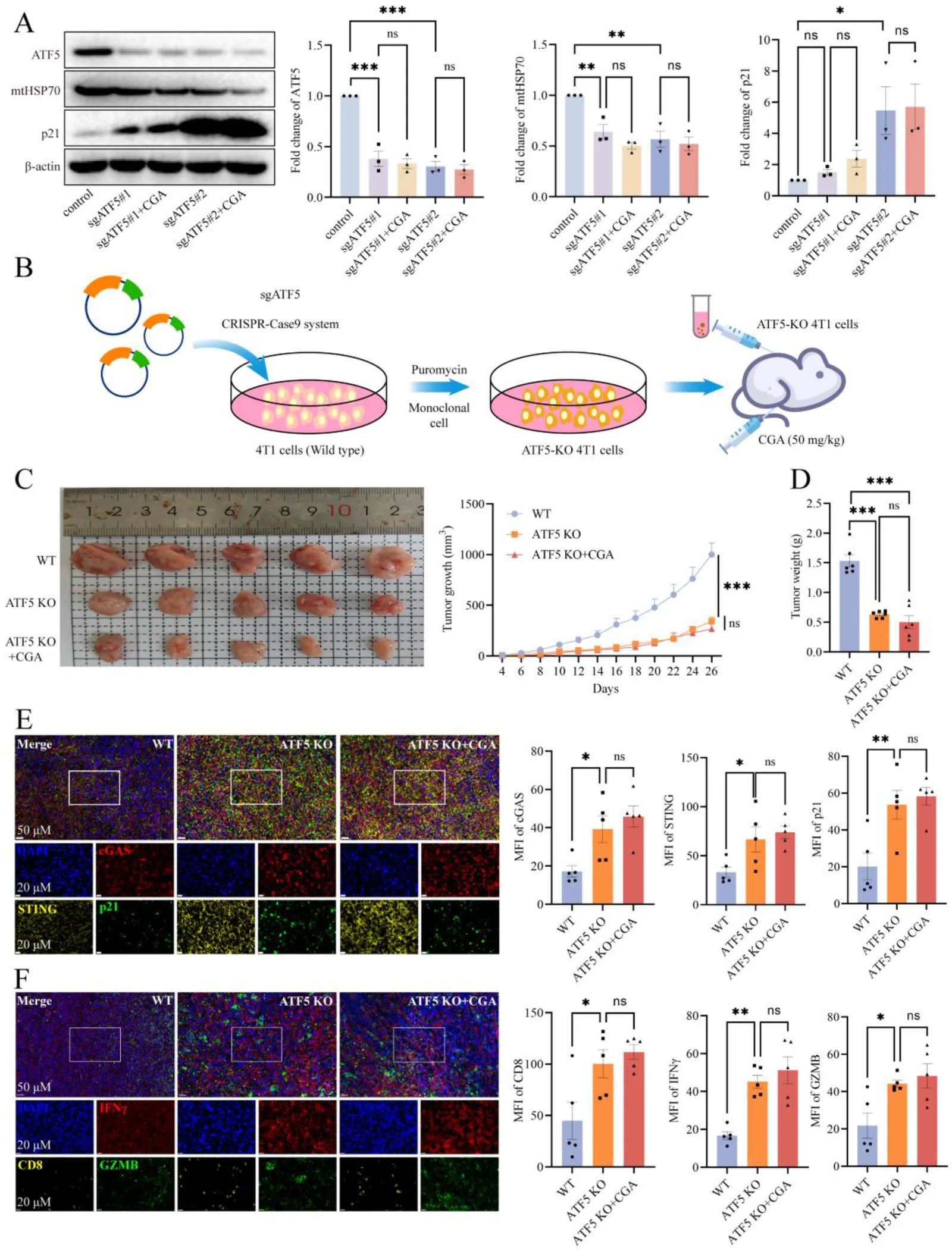
** Knockout of ATF5 inhibited tumor growth and affected ATF5 downstream signaling cascade reaction. (A)** Western blotting detection of ATF5, mtHSP70 and p21 in 4T1 cells with knockout of ATF5. **(B)** The flow diagram of establishing tumor bearing mice model with ATF5-KO 4T1 cell line. **(C)** The excised tumor and tumor volume of ATF5-knockout 4T1-tumor tissues. **(D)** The tumor weight of ATF5-knockout 4T1-tumor tissues.** (E)** Representative images of immunofluorescence staining for cGAS, STING and p21 in the wild-type and ATF5-knockout 4T1-tumor tissues. **(F)** Representative images of immunofluorescence staining for CD8, IFNγ and GZMB in the wild-type and ATF5-knockout 4T1-tumor tissues. Scale bar, 50 μm and 20 μm. Data are presented as mean ± SEM (n = 3 or 5 or 6), analyzed using one-way ANOVA. *p < 0.05, **p < 0.01, ***p < 0.001, ns, no significance. MFI: mean fluorescence intensity; IFNγ: interferon-γ; GZMB: granzyme B.

To investigate the role of ATF5 suppression by CGA, ATF5 expression was knocked out in the 4T1 cell line using the CRISPR-Cas9 system. Two sgRNAs targeting the ATF5 gene were employed to ensure effective knockout, establishing the ATF5-KO 4T1 cell line. As shown in **Fig. 7A**, both sgATF5s successfully knocked out ATF5 at the protein level, with no significant effect observed upon CGA treatment. Additionally, ATF5 knockout induced p21 expression, suggesting that ATF5 inhibition is linked to tumor differentiation. Subsequently, the ATF5-KO 4T1 cell line was used to establish a tumor-bearing mouse model (**Fig. 7B**). Tumor growth was significantly inhibited by 65.9% in the ATF5-KO 4T1 group compared to the wild-type (WT) group (ATF5-KO 4T1 vs. WT, n = 6, p < 0.001) (**Fig. 7C**), indicating that ATF5 inhibition is positively correlated with tumor suppression. However, CGA treatment (50 mg/kg, i.p. for 26 days) did not show a significant effect in the ATF5-KO 4T1 group compared to the ATF5-KO 4T1 group without CGA treatment (ATF5-KO 4T1+CGA vs. ATF5-KO 4T1, n = 6, ns) (**Fig. 7C**). Tumor weight was reduced by 58.8% in the ATF5-KO 4T1 group and by 67.1% in the ATF5-KO 4T1+CGA group compared to the WT group (vs. WT, n = 6, p < 0.001, 0.001) (**Fig. 7D**). No significant difference in tumor weight was observed between the ATF5-KO 4T1 and ATF5-KO 4T1+CGA groups, suggesting that CGA exerts its anti-tumor effects via ATF5 inhibition. Immuno-fluorescence assays revealed that ATF5 knockout induced the expression of cGAS, STING, and p21 in tumor tissues (ATF5-KO 4T1 vs. WT, n = 5, p < 0.05, 0.05, 0.01), but no further enhancement was observed in the ATF5-KO 4T1+CGA group (ATF5-KO 4T1+CGA vs. ATF5-KO 4T1, n = 5, ns, ns, ns) (**Fig. 7E**). Flow cytometric analysis of immune responses in tumor tissue showed that compared to the WT group, the MFI of CD8, IFN-γ, and GZMB was significantly higher in the ATF5-KO 4T1 group (ATF5-KO 4T1 vs. WT, n = 5, p < 0.05, 0.01, 0.05) (**Fig. 7F**). CGA treatment under ATF5 knockout enhanced the expression of CD8, GZMB, and IFN-γ, but no significant difference was observed compared to the ATF5-KO 4T1 group. Based on these findings, ATF5 may be a potential drug target in cancer therapy, and the anti-tumor effects of CGA are, at least in part, mediated through the inhibition of ATF5. In summary, CGA treatment appears to delay tumor progression, potentially by downregulating ATF5 and activating the cGAS-STING-mediated immune response.

## Discussion

This study identifies a previously unrecognized mitochondrial signaling cascade through which CGA activates antitumor immunity. CGA reduces cellular ATP and OXPHOS levels in cancer cells, downregulates TFAM transport into mitochondria by inhibiting the ATF5-mtHSP70 system, and promotes mtDNA leakage, which activates the cGAS-STING signaling pathway and increases NK cell and CTL infiltration.

Intracellular and extracellular ATP levels, along with cellular metabolism, influence tumor progression and immune surveillance 27. High ATP production in cancer cells is associated with drug resistance 28, suggesting that downregulating ATP levels could suppress tumor growth. In this study, CGA administration reduced ATP content and decreased OCR during OXPHOS, consistent with our previous findings 20. Moreover, the attenuation of COXV expression following CGA treatment indicates inhibition of mtDNA function. Previous studies on mitochondrial ACAT1 suggest that CGA's anti-tumor effects may be closely linked to mitochondrial regulation. These observations suggest that mitochondrial regulation may represent a fundamental mechanism underlying the pleiotropic biological effects of CGA. Tumor cells exist in a highly disordered biological state characterized by sustained energy demand, uncontrolled proliferation, mitochondrial dysfunction, and loss of differentiation. We therefore speculate that CGA exerts a negentropic effect by suppressing pathological mitochondrial metabolism and restoring cellular homeostasis, thereby shifting tumor cells toward a more ordered biological state 29, 30. This concept is supported by the ability of CGA to induce tumor cell differentiation and inhibit malignant progression. Although the proposed negentropy hypothesis requires further experimental validation, it may provide a novel theoretical framework for understanding the multifaceted antitumor mechanisms of CGA.

The transcription and replication of mtDNA rely on a system involving POLRMT (mitochondrial RNA polymerase), TFB2M, and TFAM 31. Pharmacological inhibition of POLRMT suppresses mtDNA expression and OXPHOS, thereby inhibiting tumor growth 32. Previous studies have demonstrated that disruption of TFAM function impairs mitochondrial homeostasis and contributes to antitumor effects. Hu *et al*. demonstrated that TFAM loss, induced by ATM inhibition, leads to mtDNA leakage into the cytoplasm 11. In addition, LONP1-mediated TFAM degradation impairs mtDNA maintenance and suppresses pancreatic tumor progression 33, whereas TFAM deficiency has also been linked to excessive mitophagy and ferroptosis 34. These findings highlight TFAM as a central regulator of mitochondrial integrity and a potential therapeutic target in cancer. Based on the high expression of TFAM in glioma and the promising clinical activity of CGA in recurrent high-grade glioma, we investigated whether TFAM is involved in the antitumor mechanism of CGA. Our results demonstrated that CGA selectively reduced mitochondrial TFAM without altering total cellular TFAM expression, accompanied by decreased mtDNA copy number and increased cytosolic mtDNA. These findings suggest that CGA disrupts mitochondrial TFAM homeostasis, thereby impairing mtDNA maintenance and promoting mtDNA leakage, which may contribute to the subsequent activation of the cGAS-STING pathway and antitumor immune responses.

The import of TFAM is a highly coordinated process involving the TOM-TIM translocase complexes, the presequence translocase-associated motor (PAM), and the mitochondrial processing peptidase (MPP) 25, 35-37. As the ATP-dependent motor of the PAM complex, mtHSP70 drives the translocation of precursor proteins into the mitochondrial matrix and facilitates their proper folding and quality control 25, 38. Importantly, mtHSP70 is transcriptionally regulated by ATF5, a key mediator of the mitochondrial unfolded protein response (UPR^mt^) that maintains mitochondrial proteostasis under stress conditions 39. Both ATF5 and mtHSP70 have been implicated in cancer cell survival and tumor progression, and elevated ATF5 expression has been reported in multiple malignancies, including glioblastoma, melanoma, and breast cancer 26. Previous studies have highlighted the critical role of ATF5 in mitochondrial stress responses and tumor cell proliferation 40, 41. Targeting ATF5 has been suggested as a strategy to attenuate cancer stemness and progression 42, 43. CGA markedly reduced ATF5 expression, which was accompanied by decreased mtHSP70 expression and selective reduction of mitochondrial TFAM. Furthermore, ATF5 knockout significantly inhibited tumor growth, whereas CGA treatment failed to exert additional antitumor effects in ATF5-deficient tumors, indicating that ATF5 is an important mediator of the antitumor activity of CGA. Collectively, these findings identify the ATF5-mtHSP70-TFAM axis as a previously unrecognized upstream regulatory pathway through which CGA disrupts mitochondrial homeostasis. This mechanism may contribute, at least in part, to the antitumor effects of CGA by coordinating mitochondrial dysfunction. Notably, while CGA has been shown to inhibit the phosphorylation of mitochondrial ACAT1 at the Y407 residue 21, whether ACAT1 interacts with the ATF5-mtHSP70-TFAM axis or regulates mitochondrial protein homeostasis through complementary mechanisms remains unknown and warrants further investigation.

The stimulation of the cGAS-STING pathway has garnered significant attention in cancer therapy in recent years 15. However, the clinical application of STING agonists is still limited by systemic toxicity and off-target effects. Cytosolic mtDNA alone is sufficient to activate the cGAS-STING pathway, suggesting that promoting mtDNA leakage may be an effective approach to activate immune responses 44. Cui *et al*. demonstrated that reuterin, a major metabolite of *Lactobacillus reuteri*, induces pyroptosis through mtDNA-mediated STING activation in hepatocellular carcinoma 10. Similarly, dimethyl fumarate enhanced anti-tumor immunity by promoting mtDNA release and activating the cGAS-STING pathway in cervical cancer 45. In the present study, CGA-induced mtDNA leakage activated the cGAS-STING pathway, resulting in increased expression of type I interferons and activation of immune response. Considering the marked molecular heterogeneity among tumor types, it is not unexpected that the magnitude of CGA-induced responses varies across different cancer cell lines. Although activation of STING signaling can lead to the secretion of type I interferons, which may in turn activate the STAT1/3 pathway and upregulate PD-L1 expression 46, our previous study showed that CGA attenuates IFN-γ-induced PD-L1 expression by inhibiting STAT1 phosphorylation and IRF1 expression 22. Therefore, although STING activation has been reported to promote PD-L1 expression in certain contexts, CGA may counterbalance this effect through its inhibitory regulation of the STAT1-IRF1 signaling pathway. Furthermore, depletion of nuclear PD-L1 has been linked to the upregulation of STING transcription and induction of p21 expression 47. In the ATF5 knockout tumor-bearing mouse model, the expression of cGAS and STING in tumor tissues was significantly enhanced, supporting the notion that inhibition of ATF5 facilitates mtDNA-mediated activation of the cGAS-STING pathway.

Activation of the STING pathway triggers an innate immune response, which was evidenced by increased infiltration of innate immune cells in tumor tissues following CGA administration in two tumor-bearing mouse models (**Fig. 6A**). Furthermore, CGA consistently promoted CTL infiltration, as previously described 22. Currently, STING agonists are being investigated in Phase I/II clinical trials, either alone or in combination with immune checkpoint inhibitors (ICIs), such as anti-PD-1 and anti-PD-L1 15. These combination therapies have shown promise in boosting anti-tumor immunity and have been well tolerated in patients 48, 49. Our prior research demonstrated that CGA effectively enhances the therapeutic efficacy of anti-PD-1 antibodies in both “hot” and “altered” tumor-bearing mouse models 22. Therefore, the STING activation observed in this study provides a mechanistic explanation for CGA's promotion of anti-tumor immunity. Systemic administration of STING agonists can lead to excessive pro-inflammatory cytokine production and cytokine release syndrome 50. However, the lack of a significant increase in systemic IFN-α and IFN-β levels in serum following CGA treatment in this study suggests that STING activation by CGA is localized primarily within tumor tissues. Indeed, the safety of CGA administration has been demonstrated in Phase I clinical trials and previous studies 19, 20. Given the structural differences between murine and human STING, direct stimulation of STING in pre-clinical murine models may not always correlate with effective activation in humans 51. Therefore, stimulating STING through an upstream, endogenous pathway could offer an effective and safer approach. But the present study mainly evaluated the short-term antitumor effects of CGA, the consequences of long-term cGAS-STING activation were not investigated. Thus, CGA may indirectly activate the cGAS-STING pathway by promoting mtDNA leakage, thereby enhancing antitumor immunity.

## Conclusion

**Figure 8:**
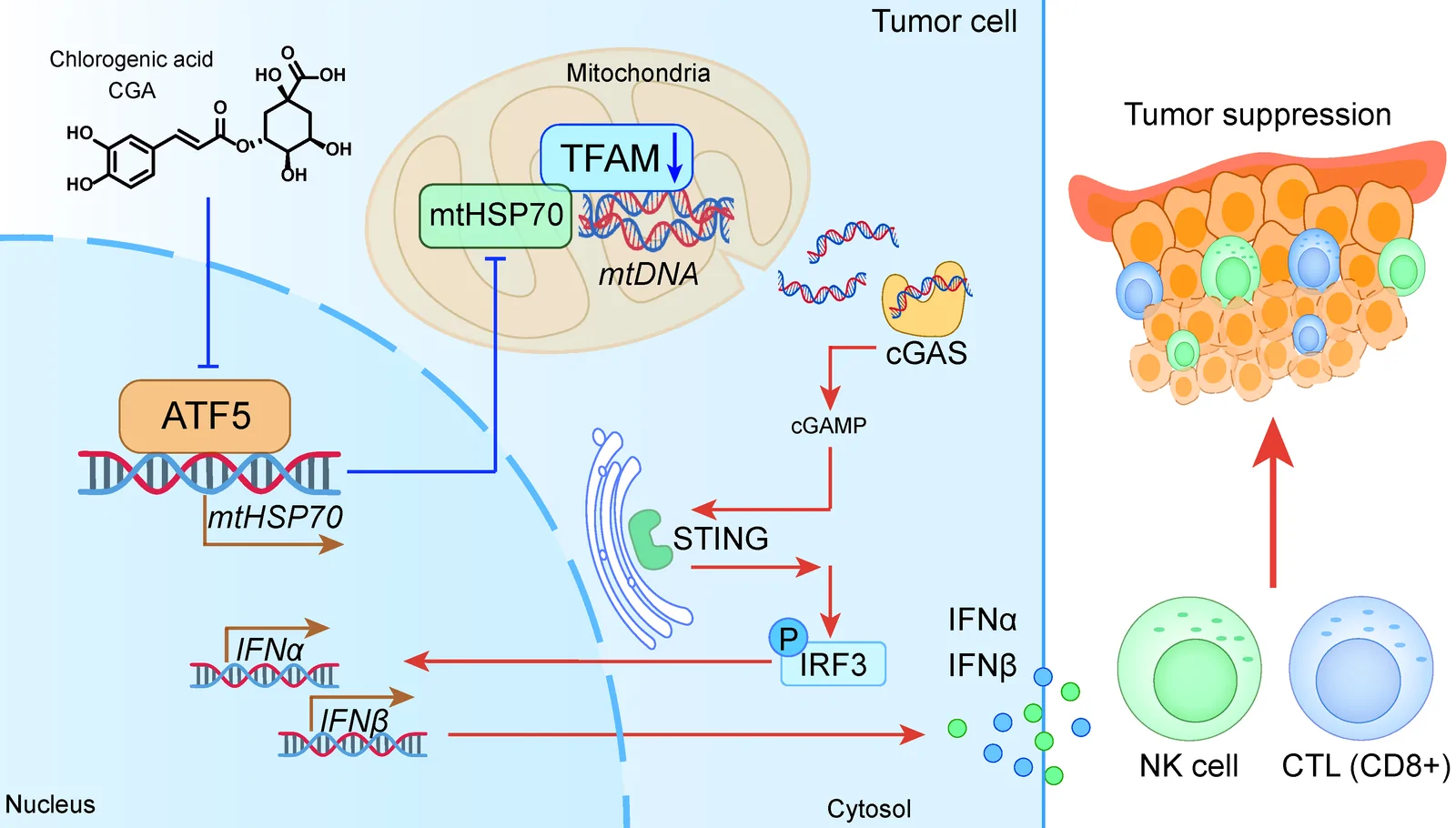
CGA promotes anti-tumor immune response by inhibiting ATF5-mtHSP70 system and activating cGAS-STING signal transduction cascade.

In this study, we demonstrate that CGA disrupts the ATF5-mtHSP70-TFAM axis, thereby reducing mitochondrial TFAM, promoting mtDNA leakage into the cytoplasm, activating the cGAS-STING pathway, enhancing NK cell and CTL infiltration, and ultimately suppressing tumor growth (**Fig. 8**). Our previous studies characterized several independent biological activities of CGA, whereas the present study provides a unified mitochondrial signaling framework that mechanistically links these previously reported phenotypes. Future studies should further elucidate how CGA regulates ATF5 activity and determine whether additional mitochondrial targets cooperate with the ATF5-mtHSP70-TFAM axis to regulate mitochondrial homeostasis and antitumor immunity. Collectively, this work provides mechanistic insight into how mitochondrial homeostasis can be exploited to coordinate tumor differentiation and antitumor immunity, highlighting the therapeutic potential of CGA for cancer treatment.

## Supplementary Material

Supplementary figures and table.

## Abbreviations

ATP: adenosine triphosphate
ATF5: activating transcription factor 5
CSCs: cancer stem cells
CDN: cyclic dinucleotide
CGA: chlorogenic acid
COXV: cytochrome c oxidase subunit V
cGAMP: cyclic GMP-AMP
cGAS‌: cyclic GMP-AMP synthase
CTLs: cytotoxic T lymphocytes
dsDNA: double-stranded DNA
ETC: electron transport chain
GBM: glioblastoma
GZMB: granzyme B
IFNs: type I interferons
IFNα: interferon α
IFNβ: interferon β
IFNγ: interferon γ
ICIs: immune checkpoint inhibitors
mtDNA: mitochondrial DNA
mt-Co1: mitochondrial cytochrome c oxidase 1
mtND1: mitochondrial ND1
mtHSP70: mitochondrial heat shock protein 70‌
MPP: mitochondrial processing peptidase
NS: normal saline
NSCLC: non-small cell lung cancer
NK: natural killer cell
OXPHOS: oxidative phosphorylation
OCR: oxygen consumption rate
POLRMT: mitochondrial RNA polymerase
PAM: presequence translocase-associated motor
STING: stimulator of interferon genes‌
TFAM: mitochondrial transcription factor A
TFB2M: mitochondrial transcription factor B2
TOM: translocase of the outer membrane
TIM: translocase of the inner membrane
UPR^mt^: unfolded protein response
