# Supplementary material for: Chlorogenic acid promotes mtDNA leakage to enhance cGAS-STING induced anti-tumor immunity via ATF5-mtHSP70-TFAM system
Source: Int J Biol Sci. 2026 Aug 12;22(13):7277–94. doi: 10.7150/ijbs.136878 (PMC13540701; doi:10.7150/ijbs.136878)
Supplement: Supplementary file 1 [file ijbsv22p7277s1.pdf]

Chlorogenic acid promotes mtDNA leakage to enhance cGAS-STING induced anti-tumor immunity via ATF5-mtHSP70-TFAM system

Rui Li [1, 3, #, *], Ping Zhou [2, #], Chu-juan Hu [1], Ge Si [1], Ling Ren [1], Jin-jin Cui [1], Ying-ying He[1], Yan-xing Han [2], Jie Zhang [4], Wen-bin Li [5], Lu-lu Wang [1, *], Jian-dong Jiang [1, 2, *]

1. State Key Laboratory of Bioactive Substance and Function of Natural Medicines, Institute of Medicinal Biotechnology, Chinese Academy of Medical Sciences & Peking Union Medical College, Beijing, 100050, PR China;
2. Institute of Materia Medica, Chinese Academy of Medical Sciences & Peking Union Medical College, Beijing, 100050, PR China.
3. Beijing Key Laboratory of Long-acting Targeted Delivery Technology and Application for Peptide and Nucleic Acid Drugs, Institute of Medicinal Biotechnology, Chinese Academy of Medical Sciences & Peking Union Medical College, Beijing, 100050, PR China.
4. Jiuzhang Biochemical Engineering Science and Technology Development Co., Ltd, Chengdu, Sichuan, 610041, PR China.
5. Department of Neuro-oncology, Cancer Center, Beijing Tiantan Hospital, Capital Medical University, Beijing, 100070, PR China.

Supplementary Data

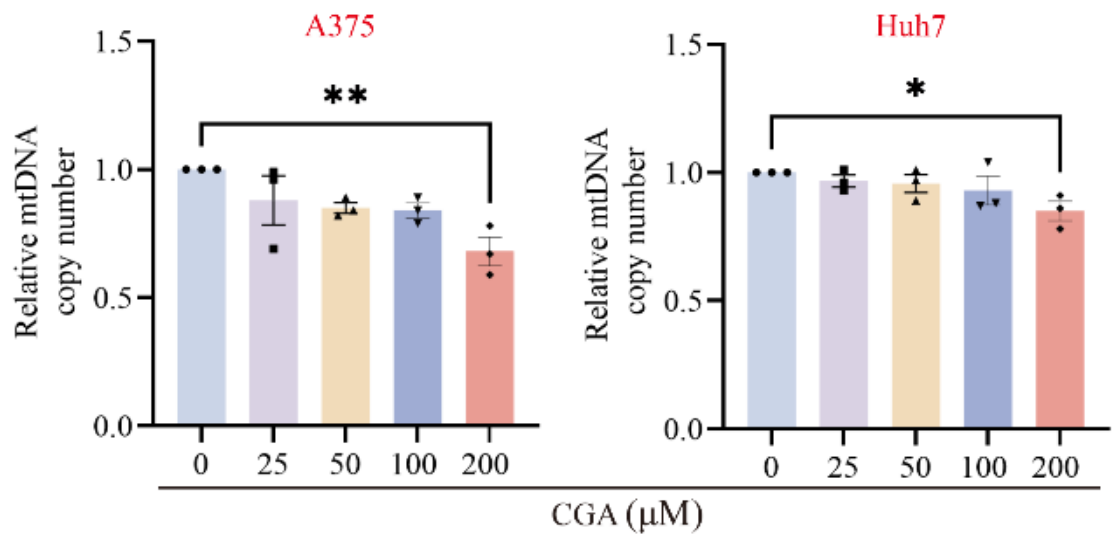

Fig.S1 CGA modulated mitochondrial functions in tumor cells. Human melanoma cell line A375 and human hepatoma cell line Huh7 were treated with CGA (0, 25, 50, 100, 200 μM) for 24 h. qRT-PCR quantification of the copy number of mtDNA in A375 and Huh7 cells. Data are presented as mean ± SEM (n=3), analyzed using one-way ANOVA. *$p < 0.05$，**$p < 0.01$.

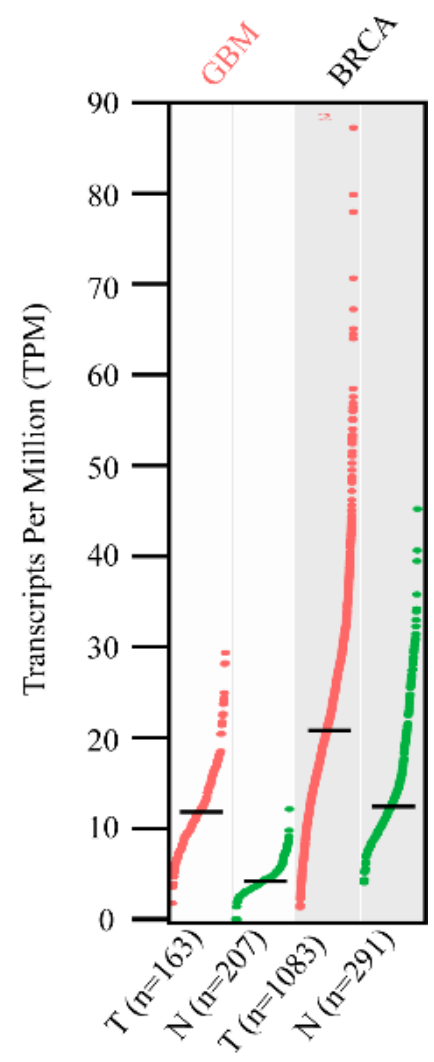

Fig. S2 TFAM had high expression in glioblastoma patients. GEPIA data indicated the high expression of TFAM in glioblastoma compared to normal group.

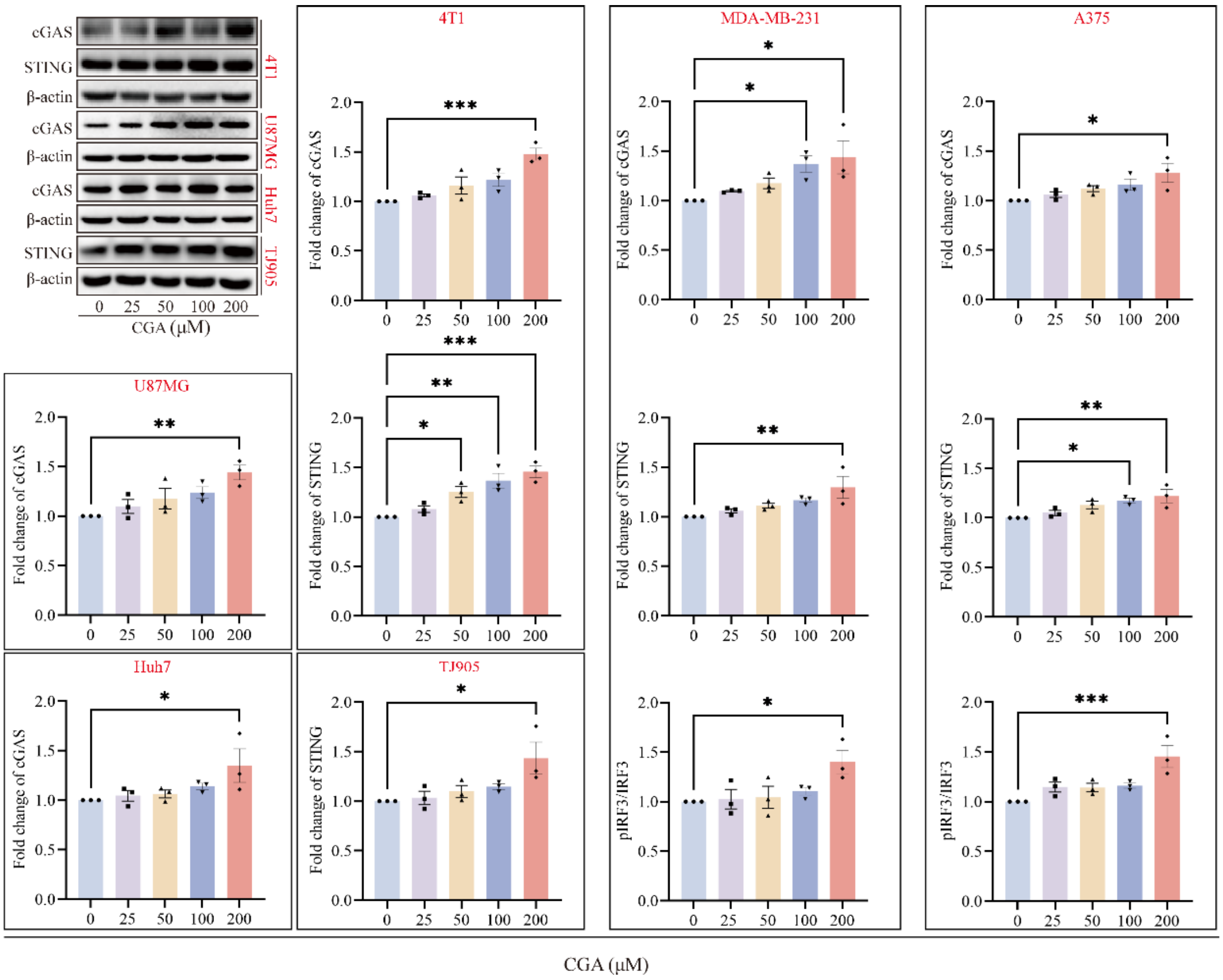

Fig.S3 CGA stimulated cGAS/STING signaling pathway. Tumor cells were treated with CGA (0, 25, 50, 100, 200 μM) for 24 h. The semi-quantitative protein expression of cGAS, STING and pIRF3/IRF3 in the indicated cell lines. Data are presented as mean ± SEM (n=3), analyzed using one-way ANOVA. *$p < 0.05$， **$p < 0.01$， ***$p < 0.001$.

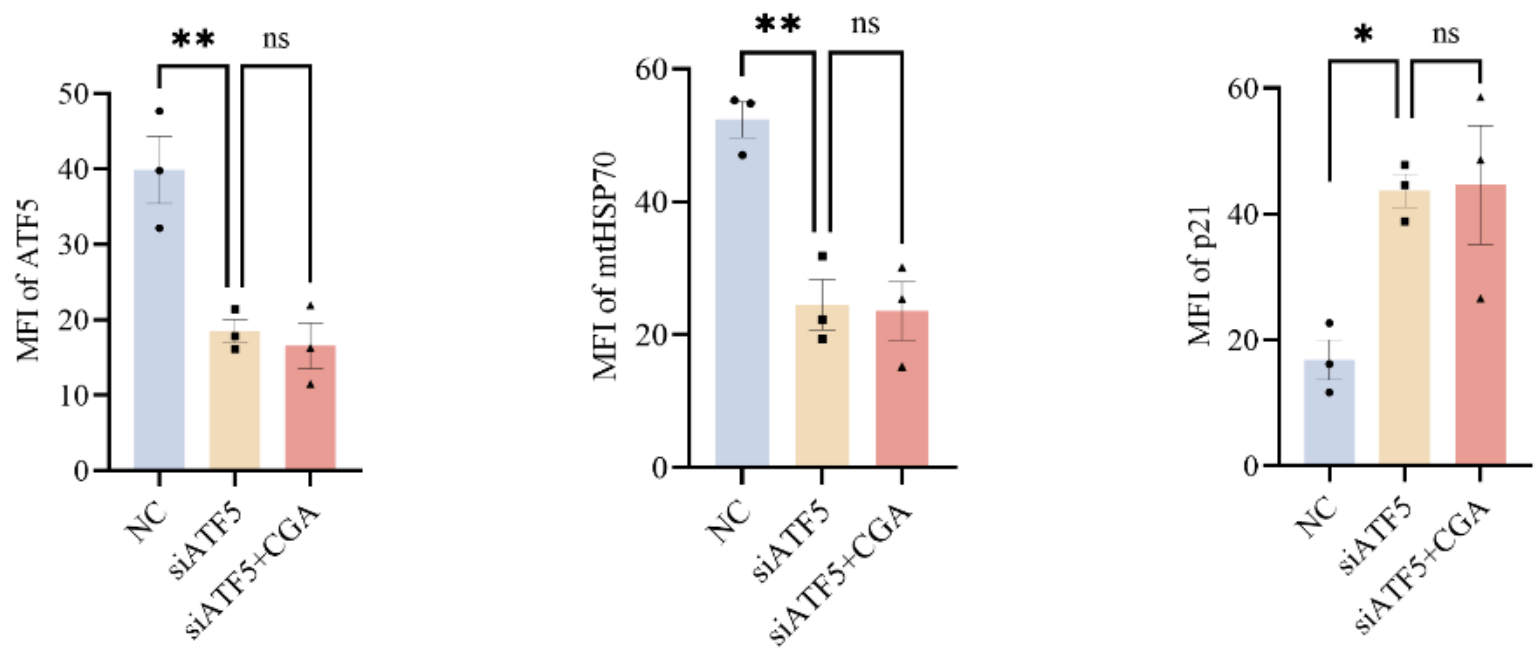

Fig.S4 The quantified mean fluorescence intensity of ATF5, mtHSP70 and p21 in MDA-MB-231 cells with knockdown of ATF5. Data are presented as mean ± SEM (n=3), analyzed using one-way ANOVA. *$p < 0.05$， **$p < 0.01$，ns, no significance. MFI: mean fluorescence intensity.

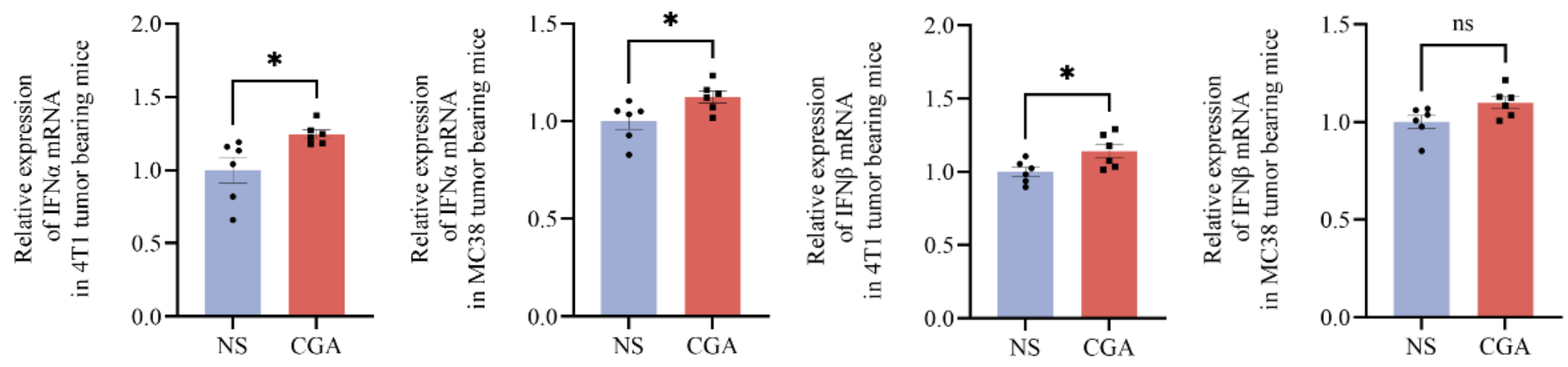

Fig.S5 CGA upregulated IFNα and IFNβ gene expressions *in vivo*. Quantification of mRNA levels of IFNα and IFNβ in the tumors of Balb/c and C57BL/6N mice. Data are presented as mean ± SEM (n= 6), analyzed using Student's t-test. *p < 0.05, ns, no significance.

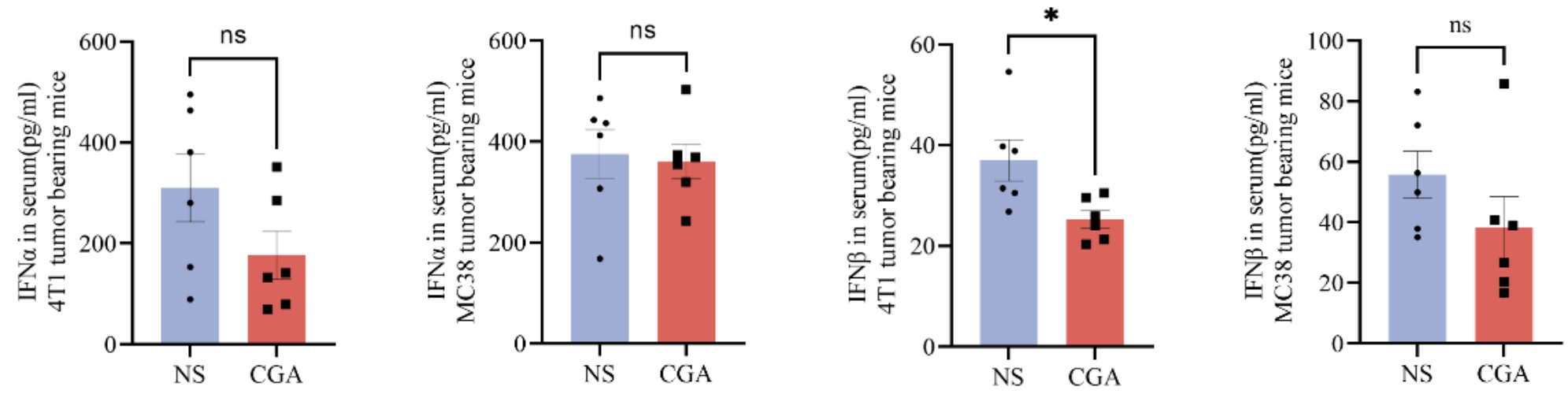

Fig.S6 CGA did not increase the serum IFNα and IFNβ concentration. The levels of IFNα and IFNβ in the serum of 4T1 and MC38 tumor bearing mice. Data are presented as mean ± SEM (n = 6), analyzed using Student's t-test. *p < 0.05, ns, no significance.

Table S1. Primer sequences for qRT-PCR

Gene name	Forward	Reverse
homo		
GAPDH	GGTGAAGGTCGGAGTCAACG	TGGGTGGAATCATATTGGAACA
ATF5	GGACCGCAAGCAAAAGAAGA	CTCGATGAGCAGGTCCTTGA
mtHSP70	TCTTGTGGGTGGCATGACTA	GTGACATCAAGGAGCAGCAC
IFNα	TCTGCACCGAACTCTACCAG	TTCTGCTCTGACAACCTCCC
IFNβ	ACGCCGCATTGACCATCTAT	GCTCATGAGTTTTCCCCTGG
Mt-COI	TTAGCTGACTCGCCACAGTC	GGCCACCTACGGTGAAAAGA
18s rDNA	CTTAGAGGGACAAGTGGCG	ACGCTGAGCCAGTCAGTGTA
HGB	GTGCACCTGACTCCTGAGGAGA	CCTTGATACCAACCTGCCCAG
ND1	CCCTAAAACCCGCCACATCT	GAGCGATGGTGAGAGCTAAGGT
murine		
Gapdh	CTCCCACTCTTCCACCTTCG	TAGGGCCTCTCTTGCTCAGT
Atf5	TGGGCTGGCTCGTAGACTATGG	TCCACCCGCTCAGTCATCCAAT
mtHSP70	TGTCCGTGCCTCCAATGGTGAT	CCTTAGTGGCCTGTCGCTGTGA
Ifnα	TTCTGCAATGACCTCCACCA	ACTTCTGCTCTGACCACCTC
Ifnβ	CCACCAGCAGACAGTGTTTC	GAAGATCTCTGCTCGGACCA
Mt-COI	GCCCCAGATATAGCATTCCC	GTTCATCCTGTTCCTGCTCC
18s rDNA	CTAGCTCATGTGTCAAGACCCTC TT	GCCAGCACGTTTCTCTCGTT
